# Experimental and numerical assessment of calcium hydroxide dosage on the seepage performance of Yellow River sediment-based alkali-activated materials for embankment seepage control

**DOI:** 10.1371/journal.pone.0362361

**Published:** 2026-11-13

**Authors:** Liwei Han, Jiayu Li, Mingkai Liu, Xinyuan Yao, Te Wang

**Affiliations:** 1 School of Water Conservancy, North China University of Water Resources and Electric Power, Zhengzhou, China; 2 Henan Key Laboratory of Water Environment Simulation and Treatment, Zhengzhou, China; Coimbatore Institute of Technology, INDIA

## Abstract

To investigate the effect of calcium hydroxide (Ca(OH)_2_) dosage on the seepage performance of Yellow River sediment-based alkali-activated materials, Yellow River sediment from the Huayuankou reach was used as the main precursor in a composite NaOH–Ca(OH)_2_ activation system. The permeability coefficient was systematically examined at Ca(OH)_2_ dosages of 2.5%–15.0% (by mass of dry sediment) and curing ages of 3, 7, and 28 days, and low-field nuclear magnetic resonance (LF-NMR) was used to characterize the pore structure and its relationship with permeability. The permeability coefficient exhibited a pronounced nonlinear response to Ca(OH)_2_ dosage. Increasing the dosage from 2.5% to 10.0% reduced the permeability coefficient by 82.58%, 82.45%, and 69.07% at 3, 7, and 28 days, respectively, whereas a further increase to 12.5% caused a rebound of 43.48%, 48.70%, and 13.80%; beyond 12.5%, the permeability decreased again. The small-pore fractal dimension correlated negatively and significantly with the permeability coefficient (R² = 0.92, 0.96, and 0.92 at 3, 7, and 28 days), and a multivariate model linking permeability coefficient, Ca(OH)_2_ dosage, and curing age was established with R² = 0.97. Two-dimensional seepage analyses of a typical Yellow River embankment (7.02 m high) using GEO-Studio show that, relative to the unreinforced embankment, reinforcement layers prepared with 10.0% and 12.5% Ca(OH)_2_ lowered the elevation of the seepage exit point from 33.45 m to 33.30 m and 33.27 m (i.e., by 0.15 m and 0.18 m), while the unit-width seepage discharge remained almost unchanged (from 6.14 × 10 ⁻ ⁷ to 6.89 × 10 ⁻ ⁷ and 6.95 × 10 ⁻ ⁷ m³/(s·m)). This indicates a redistribution of the seepage field and a lower phreatic surface, rather than a deterioration in impermeability. The results provide a reference for the resource utilization of Yellow River sediment in embankment seepage control.

## 1. Introduction

The Yellow River, one of the most sediment-laden rivers in the world, has long been characterized by the natural regime of low water discharge and high sediment load, together with intensive human activities, resulting in persistent problems in its lower reaches, including sediment deposition, riverbed aggradation, altered flood conveyance capacity, and increased flood risk [1]. Long-term hydrological analysis further indicates that human activities have exceeded climate change in driving variations in runoff and sediment load in the Yellow River Basin, highlighting that Yellow River sediment is not merely a product of natural erosion and transport, but a complex issue closely associated with basin development, ecological disturbance, and engineering regulation [2]. Therefore, achieving the high-value and low-carbon utilization of Yellow River sediment while mitigating sediment-related risks has become an urgent task in the context of ecological protection and high-quality development in the Yellow River Basin.

Existing studies on Yellow River sediment utilization have mainly focused on traditional building materials and engineering filling applications. Representative studies have demonstrated its feasibility in embankment construction materials, cemented backfill, Engineered Cementitious Composites (ECC) repair materials, ultra-high-performance concrete (UHPC) fine aggregate replacement, sintered bricks, and cement-based modification [3–8]. These studies confirm that Yellow River sediment can be incorporated into engineering material systems; however, most current approaches remain limited to aggregate substitution, bulk filling, or ordinary cement-based modification, and still face problems such as low value-added utilization, high firing energy consumption, or insufficient overall performance. One important reason is the relatively low reactivity of Yellow River sediment in its natural state. Mechanical activation studies have shown that sediment activation has an optimal grinding range rather than following a simple “the finer, the better” pattern [9]. In this context, alkali activation has gradually become an attractive route for the high-value utilization of Yellow River sediment because of its low-carbon potential, resource efficiency, and strong capacity for structural regulation.

General studies on alkali-activated materials have shown that their performance is governed by precursor chemistry, amorphous phase content, particle size distribution, activator composition, liquid-to-solid ratio, and curing regime [10–12]. In addition, the environmental sustainability of alkali-activated materials depends not only on precursor sources but also strongly on the activator system [13]. Appropriate nanomaterial incorporation and curing strategies can further regulate compactness, pore structure, and strength development [14,15]. These findings provide a theoretical basis for activating and regulating Yellow River sediment through alkali activation. Based on these understandings, researchers have begun to directly incorporate Yellow River sediment into alkali-activated and geopolymer systems. Previous studies have shown that Yellow River sediment, owing to its quartz, feldspar, and relatively high SiO_2_ and Al_2_O_3_ contents, can serve as a viable precursor for alkali-activated cementitious materials [16]. The effects of different activators, including Ca(OH)_2_, NaOH, and sodium silicate, on strength development, setting behavior, gel formation, and pore structure have also been systematically examined [17]. Ball milling combined with alkali activation has been reported to improve sediment reactivity and enhance the strength and water resistance of flood-control stones [18], while optimization of alkali equivalent and activator modulus can significantly affect the densification and mechanical performance of Yellow River sediment-based ecological cementitious materials [19]. In addition, synergistic use of Yellow River sediment with red mud and slag has shown promising potential in geopolymer systems [20,21]. Overall, research on Yellow River sediment-based alkali-activated materials has progressed from feasibility verification to activation-condition optimization and mechanism interpretation.

From the perspective of seepage control, alkali-activated materials are attractive because their hydraulic conductivity is typically one to two orders of magnitude lower than that of ordinary Portland cement-based materials, and because their permeability is governed mainly by the pore size distribution and pore connectivity rather than by the total porosity. Both the activator chemistry and the precursor fineness can be adjusted to refine the pore network and to obtain a low, controllable permeability, as also reported for alkali-activated systems in general [22,23]. These characteristics match the requirements of embankment seepage control, where a low-permeability layer is expected to lower the phreatic surface and the exit gradient within the downstream slope and thus to improve the resistance of the embankment to seepage-induced damage. For Yellow River sediment-based systems specifically, however, previous studies have focused mainly on workability, setting time, compressive strength, water resistance, and reaction mechanisms [16–21], and permeability data remain scarce. For example, Wang et al. [16] and Zhang et al. [19] showed that alkali-activated Yellow River sediment can develop a dense matrix under optimized activation, but the corresponding permeability coefficients were not reported; Song et al. [18] reported improved water resistance of alkali-activated flood-control stones after ball milling, which indirectly suggests a refined pore structure, but permeability was again not quantified.

By contrast, studies on alkali-activated materials in general have increasingly emphasized the intrinsic relationship among pore structure, transport behavior, and service performance [22–26]. Previous work has shown that transport-related properties such as chloride resistance, carbonation resistance, capillary absorption, and permeability are closely associated with structural densification, pore refinement, and microstructural evolution. Nevertheless, the pore structure–permeability relationship has not yet been fully clarified in Yellow River sediment-based systems.

Accordingly, this study used Yellow River sediment as the main precursor and adopted a NaOH–Ca(OH)_2_ composite activation system to systematically investigate the permeability evolution of Yellow River sediment-based alkali-activated materials under different Ca(OH)_2_ dosages and curing ages. Low-field nuclear magnetic resonance was further employed to characterize pore structure evolution and clarify its relationship with permeability. In addition, an engineering case study combined with numerical seepage simulation was conducted to verify whether the permeability variation identified at the laboratory scale could show trend consistency at the engineering scale. The aim of this study is to provide a theoretical basis for the development and application of Yellow River sediment-based alkali-activated materials for seepage control.

The contribution of this work is twofold. Methodologically, it establishes a quantitative link between the Ca(OH)_2_ dosage, the small-pore fractal dimension obtained from LF-NMR and the permeability coefficient of an alkali-activated Yellow River sediment system, and expresses that link as a dosage–age–permeability relationship; the dosage range in which the permeability reaches a minimum is identified and is explained through the pore size distribution rather than through the total porosity. In application, the laboratory parameters are carried over to a two-dimensional seepage model of an actual embankment section, so that the influence of a sediment-based reinforcement layer on the phreatic surface, the exit point and the discharge can be examined at the engineering scale — an assessment that has not previously been reported for Yellow River sediment-based alkali-activated materials. The remainder of this paper is organized as follows. Section 2 introduces the materials and experimental methods, including permeability testing, low-field nuclear magnetic resonance analysis, and engineering-scale numerical seepage simulation. Section 3 presents the results and discussion, with emphasis on the effects of Ca(OH)_2_ dosage and curing age on permeability, pore structure evolution, and the relationship between microstructure and macroscopic performance. Section 4 summarizes the main conclusions of this study.

## 2. Materials and methods

### 2.1 Materials

Yellow River sediment was collected from the Huayuankou reach in Zhengzhou, Henan Province, within the Yellow River Basin, and used as the main precursor material in this study. The sediment was yellowish-brown in color and had a loose particle structure. The particle gradation of the raw sediment was analyzed by the sieving method [27], and the particle size distribution curve is shown in Fig 1.

**Fig 1:**
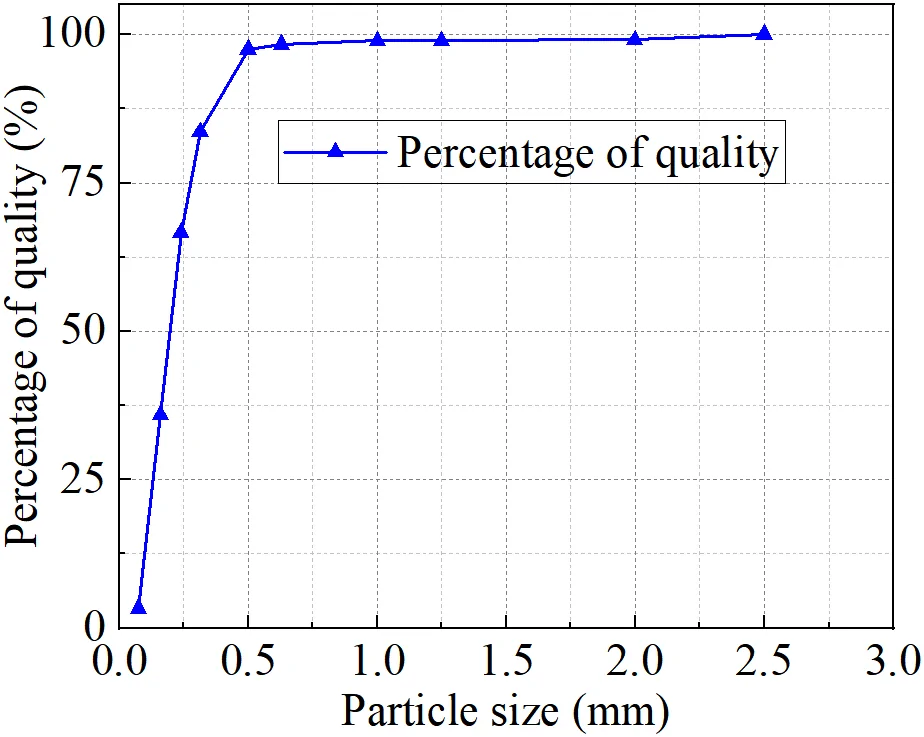
Particle size distribution curve.

Sodium hydroxide (NaOH) and calcium hydroxide (Ca(OH)_2_) were used as alkali activators in this study. Both reagents were of analytical grade and purchased from Zhengzhou Paini Chemical Reagent Factory. Deionized water was used during the entire specimen preparation process. Because of the weak cohesion between Yellow River sediment particles, the specimens were fabricated by compression molding with a controlled wet density of 2.1 g/cm³.

The Yellow River sediment used in this study was obtained from the Huayuankou reach in Zhengzhou, Henan Province. Because XRF analysis of the specific batch was not available, the chemical compositions of Yellow River sediment reported in previous studies for the same river system are compiled in Table 1 and are used here to indicate the approximate oxide composition of the precursor. The sediment is dominated by SiO_2_ (65.85–68.64 wt.%), Al_2_O_3_ (11.86–12.33 wt.%) and CaO (8.00–8.40 wt.%), with lower contents of Fe_2_O_3_ (3.25–5.14 wt.%), K_2_O (2.55–4.10 wt.%), MgO (2.05–2.63 wt.%), Na_2_O (1.30–1.57 wt.%) and TiO_2_ (0.74–1.03 wt.%) (Table 1). These values are consistent with the quartz–feldspar mineralogy and the moderate pozzolanic activity reported for Yellow River sediment [8,9,16,19]. The mean SiO_2_/Al_2_O_3_ mass ratio of approximately 5.5, together with the relatively low CaO content of about 8 wt.% (largely present as calcite, as indicated by the mineralogy reported in [8,9,19]), indicates that the sediment is a silica-rich, low-calcium precursor whose alkali activation depends on the supply of an external calcium source. This is the basis for the composite NaOH–Ca(OH)_2_ activation system adopted in the present study.

**Table 1:** Chemical composition of Yellow River sediment reported in previous studies (wt.%).

Source (sampling site)	SiO_2_	Al_2_O_3_	Fe_2_O_3_	CaO	MgO	K_2_O	Na_2_O	TiO_2_	Other^a^	LOI
Wang et al. [16], 2021 *Mengzhou, Jiaozuo, Henan*	66.40	12.10	4.00	8.00	2.50	4.10	1.30	1.00	ZrO_2_:0.10	0.38
Zhang et al. [8,9], 2023/2024 *Downstream riverbank of the Yellow River*	65.85	11.86	5.14	8.05	2.63	3.26	1.57	1.03	–	–
Zhang et al. [19], 2025 *Xixiayuan Reservoir, Henan*	68.64	12.33	3.25	8.40	2.05	2.55	n.r.	0.74	2.04^b^	–
Mean (range)	66.96 (65.85–68.64)	12.10 (11.86–12.33)	4.13 (3.25–5.14)	8.15 (8.00–8.40)	2.39 (2.05–2.63)	3.30 (2.55–4.10)	1.44 (1.30–1.57)	0.92 (0.74–1.03)	–	–

Values are compiled from X-ray fluorescence (XRF) analyses reported in previous studies on Yellow River sediment from the same river system, and are provided to indicate the approximate chemical composition of the precursor. The specific sediment batch used in the present study was not subjected to XRF analysis. n.r. = not reported separately. ^a^ “Other” denotes the sum of the remaining oxides not reported individually; depending on the source it may include Na_2_O, ZrO_2_, SO_3_, P_2_O_5_ and/or loss on ignition. ^b^ Reported by the original authors as “Other” (2.04 wt.%). Ref. [16]: Yellow River silt sampled at Mengzhou, Jiaozuo City, Henan Province. Refs. [8,9]: Yellow River sediment sampled from the downstream riverbank of the Yellow River, sieved through an 80 μm sieve (D_10_ = 17.18 μm, D_50_ = 57.49 μm, D_90_ = 99.94 μm); both references report identical compositions because the same sediment sample was used. Ref. [19]: Yellow River sediment sampled at the Xixiayuan Reservoir, Henan Province.

According to previous studies and relevant literature [10,16,19], the NaOH dosage in this study was fixed at 0.5% of the dry sediment mass and added in solid form, while the water content was maintained at 10%. The NaOH dosage was kept constant in all groups, and only the Ca(OH)_2_ dosage was adjusted to evaluate the effect of calcium source dosage on material properties. Six specimen groups were designed, namely SL-2.5, SL-5.0, SL-7.5, SL-10.0, SL-12.5, and SL-15.0, corresponding to Ca(OH)_2_ dosages of 2.5%, 5.0%, 7.5%, 10.0%, 12.5%, and 15.0%, respectively. The mix proportions are presented in Table 2.

**Table 2:** Mix proportions of the alkali-activated Yellow River sediment.

Category	Yellow River sediment	Calcium hydroxide dosage	Sodium hydroxide content	Water consumption
SL-2.5	87.0%	2.5%	0.5%	10%
SL-5.0	84.5%	5.0%	0.5%	10%
SL-7.5	82.0%	7.5%	0.5%	10%
SL-10.0	79.5%	10.0%	0.5%	10%
SL-12.5	77.0%	12.5%	0.5%	10%
SL-15.0	74.5%	15.0%	0.5%	10%

Specimens with different mix proportions were prepared according to Table 2. The molding assembly consisted of a hollow cylindrical mold with an inner diameter of 61.8 mm, an outer diameter of 81.8 mm, and a height of 120 mm, a solid cylindrical compression rod with a diameter of 61.7 mm and a height of 210 mm, and a cylindrical spacer with a diameter of 61.7 mm and a height of 15 mm. Based on the specimen dimensions, the specimen volume was calculated as 120 cm³, and 252 g of material was weighed for each specimen.

The specimens were prepared by compression molding in a cylindrical mold, with a diameter of 61.8 mm and a height of 120 mm. After molding, they were demolded and cured to the specified ages in an environment at 20 ± 2 °C and a relative humidity of approximately 95 ± 5% [15,19].

The sediment was collected from a publicly accessible riverbank at the Huayuankou reach. No specific permits were required for this sampling, as it did not involve protected areas, protected species or any activity restricted by law.

### 2.2 Experimental methods

#### 2.2.1 Permeability test.

To evaluate the permeability of Yellow River sediment-based alkali-activated materials, falling-head permeability tests were conducted in the laboratory [28–30]. The tests were performed according to the principles of ASTM D5084, with appropriate adjustments made to account for the characteristics of the alkali-activated specimens. The testing system consisted of combined TST-55 and TST-70 permeameters, as schematically illustrated in Fig 2.

**Fig 2:**
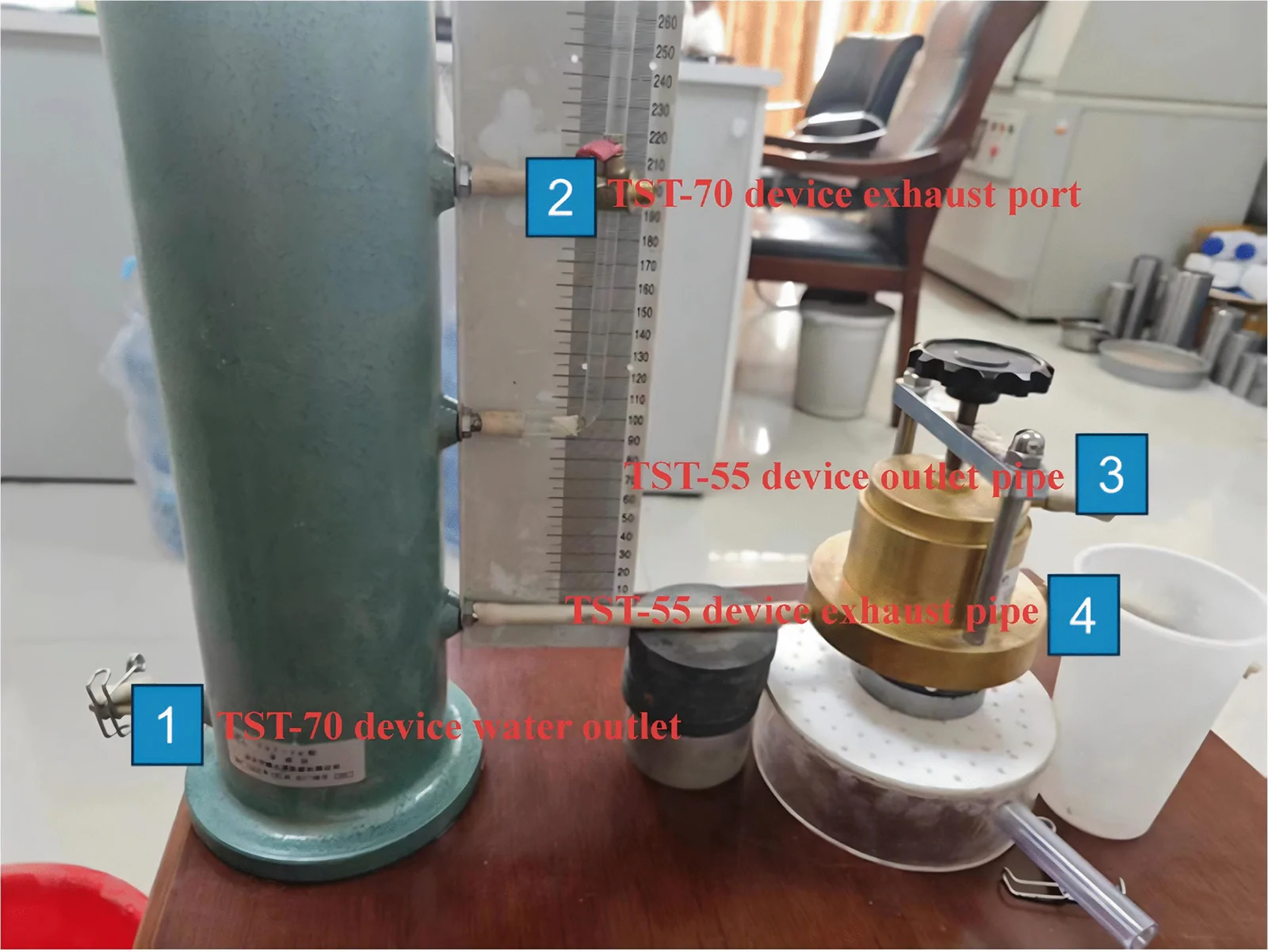
Falling-head permeability testing apparatus (Image from Han et al., 2026, distributed under a Creative Commons Attribution (CC BY 4.0) license.).

Before testing, the molded specimens were saturated to ensure that the pores were fully filled with water. After saturation, falling-head permeability tests were conducted under constant-temperature conditions. During the test, the change in water level in the standpipe was recorded over time, and the permeability coefficient of the specimen was calculated from the time interval and the corresponding hydraulic head during the head-loss process.

The permeability coefficient K was determined using the falling-head equation derived from Darcy’s law [31–33], which accounts for the relationship between the rate of hydraulic head decline in the standpipe and elapsed time.


$$\begin{array}{c} K=\frac{sl}{S(t_{\text{2}}-t_{\text{1}})}ln\frac{h_{\text{1}}}{h_{\text{2}}} \end{array}$$ (1)

where K is the permeability coefficient (m/s); S is the cross-sectional area of the specimen (m^2^); $h_{\text{1}}$ and $h_{\text{2}}$ are the initial and final hydraulic heads (m), respectively; L is the specimen height (m); and *s* is the inner cross-sectional area of the standpipe (m²).

To improve the reliability of the test results, each permeability test was repeated at least three times for each group, and the repeated measurements of the permeability coefficient showed relatively small dispersion. The permeability coefficients reported in this paper are the average values obtained from multiple tests. All permeability tests were conducted at room temperature of 20 ± 2 °C to minimize the influence of environmental factors on the test results.

Each dosage–age combination was tested on three replicate specimens, and the results are reported as mean ± standard deviation (SD). Differences between dosage groups at the same curing age were examined by one-way analysis of variance followed by Tukey’s honestly significant difference test, with differences regarded as significant at p < 0.05; the same procedure was applied when comparing curing ages within a dosage group.

Each specimen was 61.8 mm in diameter and 40 mm in height, corresponding to a volume of 120 cm³ and a mass of 252 g of dry mixture, and was tested inside its mould so that the lateral surface remained confined. Water was driven upwards through the specimen, reproducing the direction of seepage within an embankment. After saturation, the head was allowed to fall and the time and outflow were recorded at every 10 mm of head drop; five consecutive head intervals were taken as one data set, after which the supply cylinder was refilled and the procedure repeated. For each dosage–age combination the interval values were combined into a single representative value by a trimmed mean (with the largest and smallest values removed), which limits the influence of the first interval, in which saturation may not yet be complete, and of the last interval, in which the head difference is too small for a reliable reading. The standard deviation of the interval values is reported in Table 8 as an indicator of the dispersion of the measurements.

#### 2.2.2 Low-field nuclear magnetic resonance (LF-NMR) test.

A MesoMR12-120H-I permanent-magnet NMR analyser (Suzhou Niumag Corporation, China) was used, as shown in Fig 3. The instrument operates at a magnetic field strength of 0.3 ± 0.05 T, corresponding to a ¹H Larmor frequency of 12.8 MHz, and the magnet is held at 32 ± 0.01 °C. A 150 mm diameter probe coil was employed, and the centre frequency and pulse width were calibrated before each measurement session. All specimens were vacuum-saturated before testing, so that the pore space was completely filled with water.

**Fig 3:**
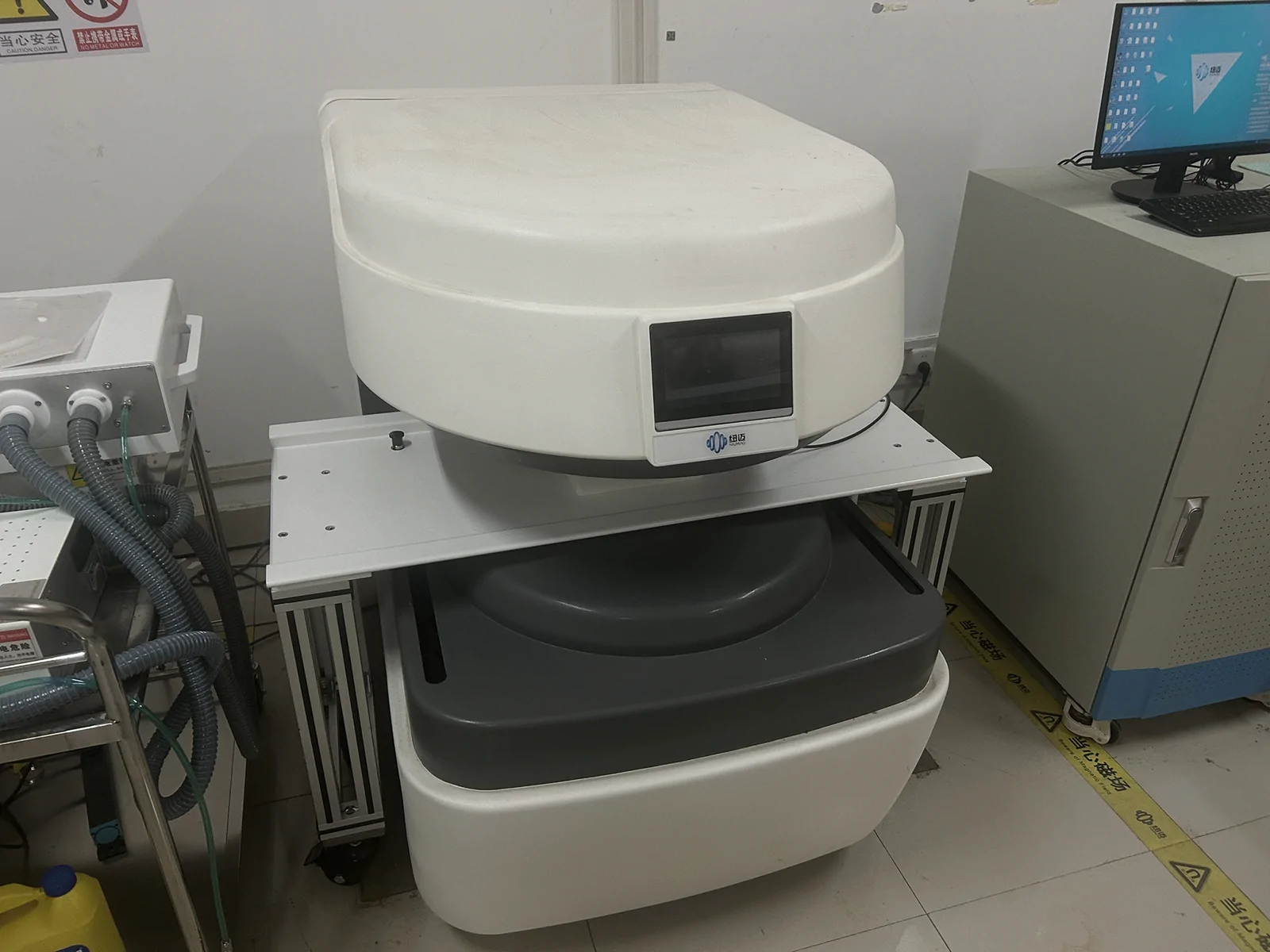
Nuclear magnetic resonance instrument (Image from Han et al., 2026, distributed under a Creative Commons Attribution (CC BY 4.0) license.).

The transverse relaxation of pore water in a saturated porous material has three contributions [34,35].


$$\begin{array}{c} \frac{\text{1}}{T_{\text{2}}}=\frac{\text{1}}{T_{\text{2S}}}+\frac{\text{1}}{T_{\text{2B}}}+\frac{\text{1}}{T_{\text{2D}}}=\rho_{\text{2}}(\frac{S}{V})+\frac{\text{1}}{T_{\text{2B}}}+D_{w}\gamma^{\text{2}}G^{\text{2}}{TE}^{\text{2}}/\text{1}\text{2} \end{array}$$ (2)

where ρ_2_ is the surface relaxivity (μm/s), S/V the surface-to-volume ratio of the pore, D the self-diffusion coefficient of water, γ the gyromagnetic ratio, G the magnetic field gradient and TE the echo spacing. For a fully saturated specimen in a homogeneous field (G ≈ 0), the bulk and diffusion terms are negligible, so that 1/T_2_ ≈ ρ_2_(S/V) = ρ_2_Fs/R, with R the pore radius and Fs a shape factor taken as 3 for the spherical-pore assumption adopted here. A surface relaxivity ρ_2_ of 3 μm/s was used, following the value established for cementitious and clay-bearing materials; the same value applies to every group because the precursor, the reaction products and the pore fluid are identical. The inverted T_2_ distributions were converted to pore radii on this basis, and the pores were classified as micropores (< 0.1 μm), small pores (0.1–1 μm), medium pores (1–10 μm) and large pores (10–100 μm). With a surface relaxivity of 3 μm/s and a shape factor of 3, the conversion reduces to *R* (μm) ≈ 0.009 T_2_ (ms), so that the four pore classes correspond to T_2_ below 11 ms, 11–111 ms, 0.11–1.11 s and above 1.11 s for pores below 0.1 μm, 0.1–1 μm, 1–10 μm and 10–100 μm, respectively.

The absolute pore radius scale depends on ρ_2_, which was not determined independently for this material. The pore size distributions are therefore used here to compare specimens prepared and measured under identical conditions rather than as absolute values, and independent verification by mercury intrusion porosimetry or gas adsorption remains to be done.

#### 2.2.3 Engineering case study and numerical seepage simulation.

An engineering case study was further employed to verify the applicability of Yellow River sediment-based alkali-activated materials in embankment seepage control. The purpose of this validation was to examine whether the permeability evolution identified at the laboratory scale could show trend consistency in seepage response at the engineering scale, rather than to provide an accurate prediction of actual engineering seepage discharge. Using the permeability parameters obtained from laboratory tests as input, the engineering analysis evaluated the influence of alkali-activated materials on embankment seepage behavior and their engineering rationality by comparing the seepage responses under the unreinforced condition and reinforced conditions with different material dosages.

The embankment is located in Jinan, Shandong Province, and represents a section of levee in the Yellow River Basin. It has a height of 7.02 m and a crest width of 13.4 m, and the embankment body mainly consists of silt. The cross-sectional profile of the embankment is shown in Fig 4.

**Fig 4:**
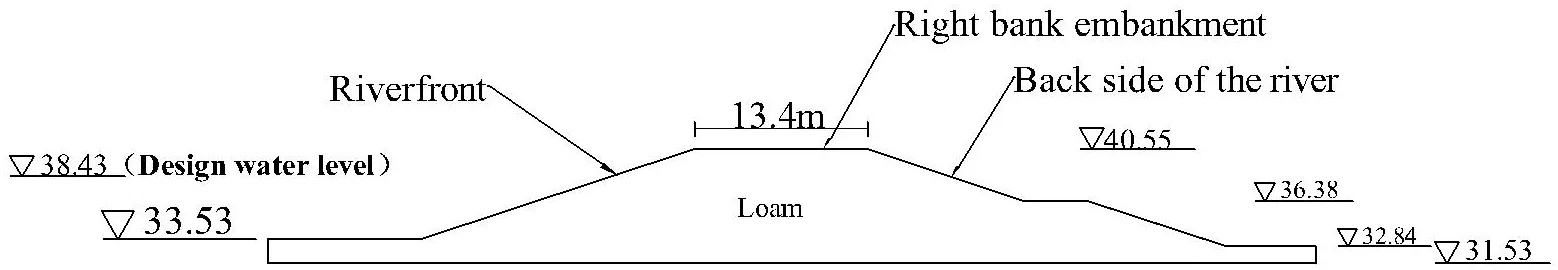
Schematic diagram of a levee model.

In the established finite element model, the computational domain included the main embankment structure, with the foundation extending a distance equal to twice the embankment height both upstream and downstream, and to a depth of 32.81 m. The embankment height was 7.02 m. Experimental observations indicated that the Yellow River sediment-based alkali-activated material had limited erosion resistance and was therefore unsuitable for direct use in the embankment body. Therefore, the material was adopted as a reinforcement layer located 30 cm beneath the upstream slope surface, with a layer thickness of 70 cm.

A two-dimensional finite element mesh was generated on the embankment cross-section, and the complete finite element model is shown in Fig 5(a). Four-node quadrilateral elements were used throughout the domain. This element type was adopted because the cross-section is geometrically regular, its material boundaries follow the slope surfaces and are therefore smooth, and the seepage problem is governed by a head field that varies gradually across most of the domain; under these conditions quadrilateral elements represent the gradients more accurately than triangular elements of comparable size, and they allow the element edges to be aligned with the reinforcement layer and with the direction of the phreatic surface, where the head changes most rapidly. The mesh was refined in and immediately around the reinforcement layer and along the two slope surfaces, and was progressively coarsened towards the lateral and lower boundaries of the foundation, where the head field is nearly uniform. The resulting mesh consists of 12,270 elements and 12,475 nodes.

**Fig 5:**
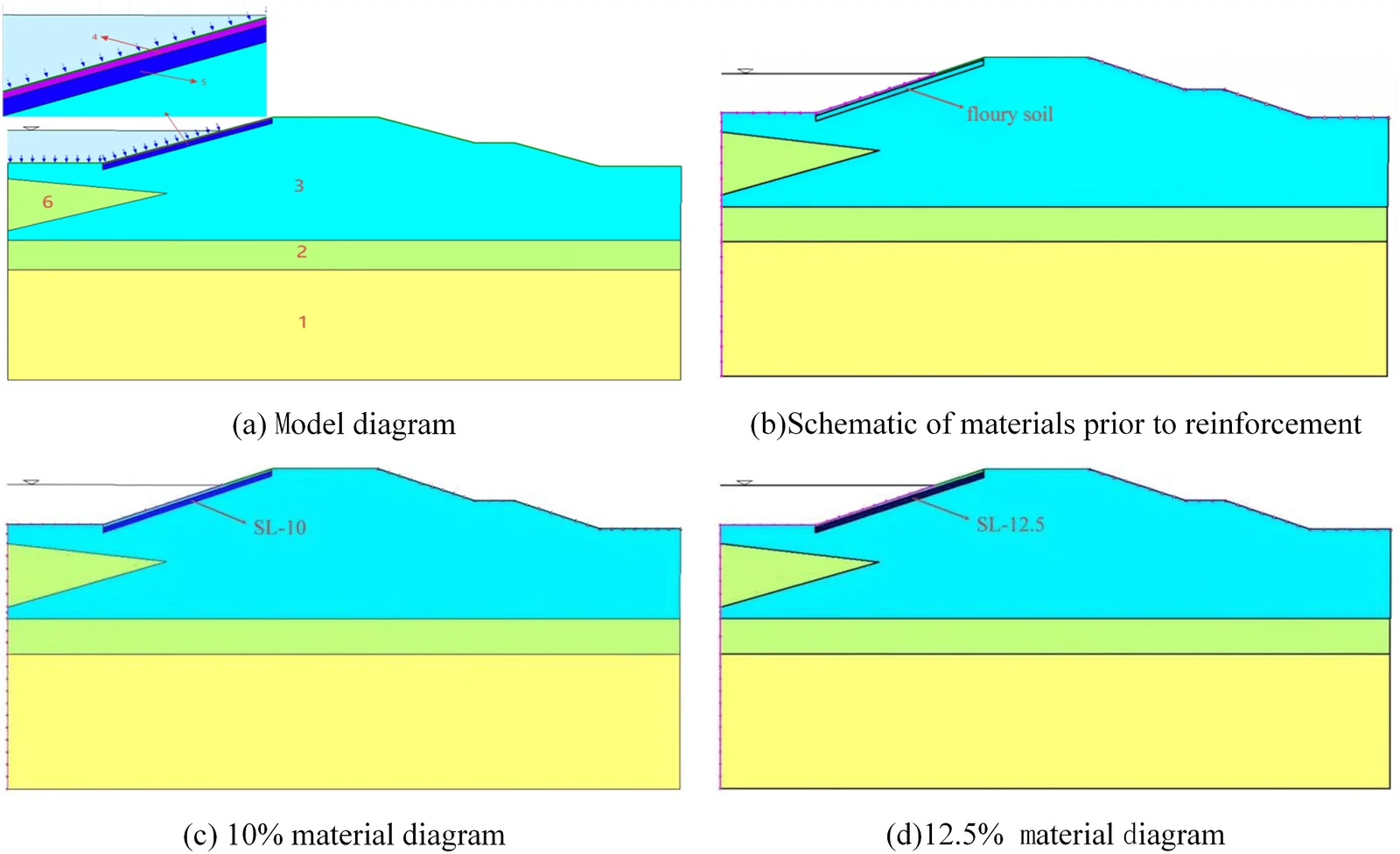
Numerical simulation model.

The model was divided into six zones according to the materials identified in the site investigation: Zone 1 is powdery clay Ⅰ; Zones 2 and 6 are powdery clay Ⅱ; Zones 3 and 4 are silt; and Zone 5 is either silt or the Yellow River sediment-based alkali-activated material, depending on the reinforcement case, as shown in Fig 5 and Table 3. The zone boundaries follow the material interfaces determined in the investigation rather than being adjusted to fit the computed solution.

**Table 3:** Material zones of the embankment model.

Region	Powdery clay Ⅰ	Powdery clay Ⅱ	Silt	SL-10	SL-12.5
1	√				
2		√			
3			√		
4			√		
5			√	√	√
6		√			

Steady-state saturated–unsaturated seepage was analysed with the SEEP/W module of GEO-Studio. The boundary conditions were prescribed as follows. The riverside (upstream) slope and the surface of the foundation were assigned a known total head, so that the design water level, at an elevation of 38.43 m — that is, a head of 4.93 m above the channel bed at 33.5 m — is imposed along the upstream side. The landside (downstream) slope was treated as a free exit (seepage-face) boundary, at which the pressure head is zero and water is free to leave the domain. The boundary between the saturated and the unsaturated zone inside the embankment was treated as a free phreatic surface, whose position was obtained by iteration between the flow solution and the zero-pressure line. The base of the foundation was treated as impermeable. The X axis of the model is taken along the channel with the positive direction downstream, and the Y axis is vertical and positive upwards. The material parameters assigned to each zone are listed in Table 4, and the calculation conditions in Table 5.

**Table 4:** Material specifications used in the numerical simulation.

Materials	Permeability coefficient *K*/(×10^–5 cm^/s)	Unit weight γ/(kN·m ⁻ ³)	φ/(°)	c/kPa	Upstream gradient
Powdery clay Ⅰ	1.74	19.4	14	35	0.33
Powdery clay Ⅱ	2.31	19	16	30	
Silt	23.1	19	25	11	
SL-10	2.52	21	39.37	179.48	
SL-12.5	2.87	21	23.45	383.31	

**Table 5:** Design conditions.

Operating conditions	River water level	
	Calculate elevation/m	Calculate the head/m
Design water level	38.43	4.93

The material parameters used for the reinforced zone were taken from the experimental results, and the permeability coefficients under different conditions were assigned according to the representative dosages identified in the laboratory tests. The corresponding material parameters and boundary conditions are summarized in Tables 4 and 5. This setup enabled a reasonable transfer of laboratory-derived parameters to the engineering scale. The selected parameters were intended to represent the relative differences in permeability behavior at the engineering scale, rather than to provide an accurate prediction of the absolute seepage discharge under actual conditions. The Ca(OH)_2_ dosages adopted for the numerical simulation (10.0% and 12.5%) were selected on the basis of the experimental results rather than arbitrarily. As shown in Sections 3.1.2 and 3.3, 10.0% is the dosage at which the permeability coefficient reaches its lowest value within the range 2.5%–12.5% at all three curing ages, and the response reverses at 12.5%, where the small-pore fractal dimension also turns over. These two dosages were therefore used in the simulation: 10.0% represents the most favourable condition found within this range and 12.5% represents its upper boundary, at which the effect of the dosage changes sign. The remaining four dosages were not included in the engineering simulation, in order to limit the number of numerical cases; their measured properties are reported in Sections 3.1–3.3 and summarized in Table 8.

Because the present numerical analysis was restricted to steady-state seepage, soil-structure interaction, deformation, and stress-seepage coupling were not considered. The reinforcement layer was represented only by Its geometric configuration and hydraulic properties.

## 3. Results and discussion

### 3.1 Permeability analysis

#### 3.1.1 Effect of curing age on permeability.

The evolution of the permeability of Yellow River sediment-based alkali-activated materials with curing age is shown in Fig 6. The permeability test results indicated that the permeability coefficient of Yellow River sediment-based alkali-activated materials varied with curing age under different Ca(OH)_2_ dosages, although the magnitude and trend of variation were not consistent among specimens with different mix proportions (Fig 6).

**Fig 6:**
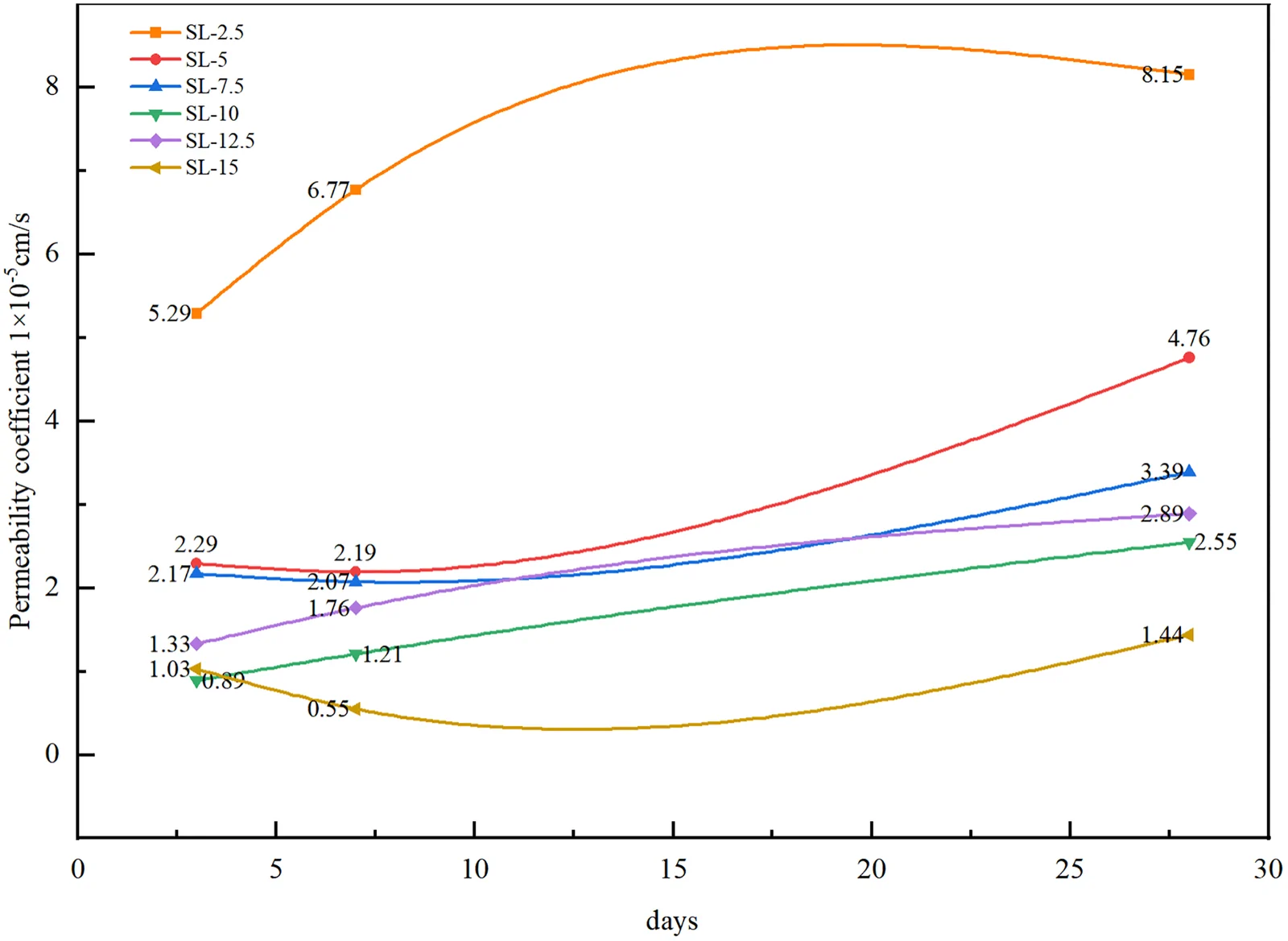
Curve showing the relationship between permeability coefficient and curing age.

Some specimens showed an increase in permeability coefficient with increasing curing age, whereas others exhibited a trend of first decreasing and then increasing. This indicates that the effect of curing age on permeability is not governed by a single factor, but is closely related to the formation of internal reaction products, pore rearrangement, and structural evolution within the material. It should be noted that, under the conditions of this study, the permeability coefficient did not exhibit a uniform monotonic trend with curing age, indicating that extending curing time alone is insufficient to precisely regulate permeability. Instead, its effect should be considered together with factors such as alkali activator dosage. The specific results are as follows:

(1) The permeability coefficients of specimens SL-5.0, SL-7.5, and SL-15.0 first decreased and then increased.

The initial decrease was attributed to the addition of calcium hydroxide, which rapidly filled specimen pores through particle flocculation and thereby reduced permeability. The subsequent increase may be associated with the later stage of the reaction, in which the formation of new reaction products slows down, while drying shrinkage and the dissolution of unreacted lime can generate new voids or microcracks that are no longer compensated by pore filling, resulting in an increase in the permeability coefficient.

(2) The permeability coefficients of specimens SL-2.5, SL-10.0, and SL-12.5 increased continuously with curing age.

#### 3.1.2 Effect of calcium hydroxide dosage on material permeability.

To further compare the permeability differences among specimens with different Ca(OH)_2_ dosages at the same curing age, the permeability coefficients at 3, 7, and 28 days were extracted and plotted, as shown in Fig 7.

**Fig 7:**
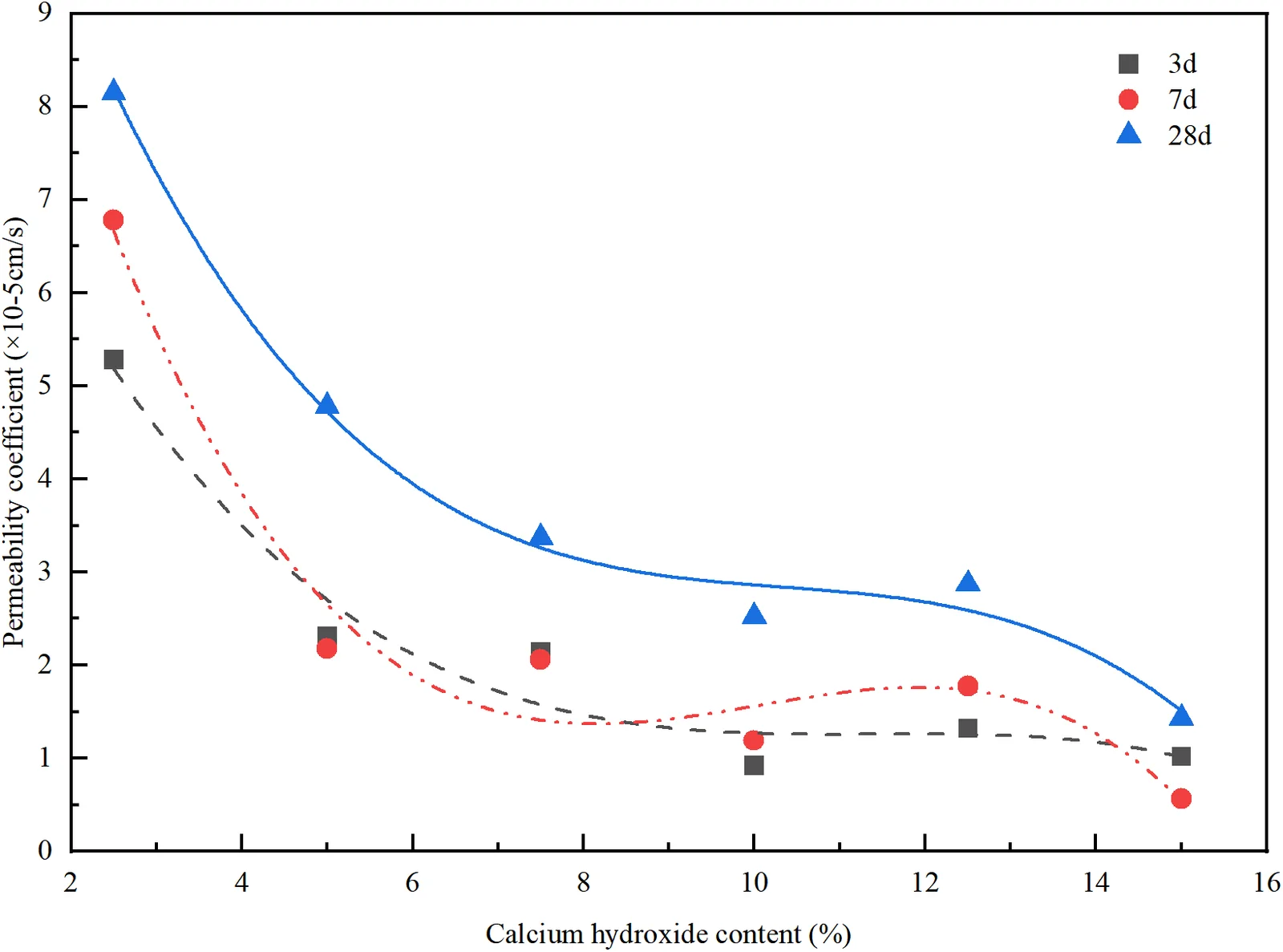
The relationship between permeability coefficient and calcium hydroxide content.

Fig 7 shows that the permeability coefficient generally decreased with increasing calcium hydroxide dosage, suggesting that calcium hydroxide improved the impermeability of the specimens, although the trend was not strictly monotonic. At 3 days, the permeability coefficient decreased by 82.58% as the dosage increased from 2.5% to 10.0%, then increased by 43.48% from 10.0% to 12.5%, and finally decreased by 22.73% from 12.5% to 15.0%, corresponding to an overall reduction of 80.68%. At 7 days, the corresponding changes were a decrease of 82.45%, an increase of 48.70%, and a decrease of 68.36%, with an overall reduction of 91.74%. At 28 days, the permeability coefficient decreased by 69.07%, then increased by 13.80%, and finally decreased by 50.17%, resulting in an overall reduction of 82.45%. Notably, at a calcium hydroxide dosage of 12.5%, the permeability coefficient deviated from the general decreasing trend observed with increasing dosage.

The reaction of Yellow River sediment with water and calcium hydroxide mainly depends on the physical and chemical interactions between the fine particles in the sediment and calcium hydroxide. When calcium hydroxide dissolves in water, it releases Ca²⁺ ions, which can neutralize the negative charges on the surfaces of sediment particles and thereby induce flocculation between particles. This flocculation increases particle aggregation and forms larger clusters, thereby reducing the porosity of the mixture and improving its impermeability. In addition, calcium hydroxide can react with silicate- and aluminate-bearing minerals in the sediment to form new compounds such as calcium silicate and calcium aluminate, which generally possess stronger cementitious properties.

The non-monotonic response can be interpreted as follows. At dosages up to about 10 wt.%, the added Ca(OH)_2_ dissolves and supplies Ca²⁺, which both neutralizes the negatively charged surfaces of the sediment particles and participates in the pozzolanic reaction with dissolved silicate and aluminate species, forming C–S–H/C–A–S–H-type gels that fill and refine the pore network; the hydraulic conductivity is thereby reduced. When the dosage exceeds the amount that can be consumed by these reactions, part of the Ca(OH)_2_ remains unreacted, the effective water available for reaction decreases, the composition of the binding phase shifts towards Ca-rich and less polymerized products with a coarser morphology, and carbonation of the excess Ca(OH)_2_ and of the C–S–H gel produces calcium carbonate accompanied by localized volume changes. These effects can reopen or enlarge part of the flow paths and thus account for the rebound in permeability observed at 12.5 wt.%. When the dosage is further increased to 15.0 wt.%, the additional calcium supply promotes the pozzolanic reaction again and the pore structure is refined once more, so that the permeability decreases. This interpretation is consistent with previous reports on Ca(OH)_2_-activated Yellow River sediment, in which the optimal Ca(OH)_2_ content was found to be 8 wt.% and dosages above 10 wt.% significantly reduced the 28-day strength owing to carbonation of the excess Ca(OH)_2_ [16], whereas a Ca(OH)_2_ content of 12.5 wt.% was reported to be optimal for sediment-based artificial flood-prevention stones [19]. The optimum dosage therefore depends on the activator system and on the target property, which is consistent with the 10.0–12.5 wt.% interval identified here for permeability. It should be emphasized that the above mechanism is inferred from the macroscopic permeability data and the LF-NMR pore structure results, supported by the literature, rather than from direct phase analysis; further mineralogical and microstructural characterization is required for verification.

#### 3.1.3 Development of a Multivariate Model for Calcium Hydroxide Dosage and Curing Age.

As shown in Section 3.1.2, the relationship between permeability coefficient and calcium hydroxide dosage could be described by a univariate cubic function. To further clarify the relationships among calcium hydroxide dosage, curing age, and permeability coefficient, a multivariate model was established on this basis, as expressed by the following equation.


$$\begin{array}{c} k=a_{\text{1}}+a_{\text{2}}n^{\text{3}}+a_{\text{3}}n^{\text{2}}+a_{\text{4}}n+a_{\text{5}}t+a_{\text{6}}nt \end{array}$$ (3)

where K denotes the permeability coefficient of the specimen (×10^−5 cm^/s), n is the calcium hydroxide dosage (%), t is the curing age (d), and $a_{\text{i}}$ are fitting parameters (i=0~6).

Through fitting analysis, a multivariate mathematical function was established to describe the relationship among permeability coefficient, calcium hydroxide dosage, and curing age, as given below.


$$\begin{array}{c} k=11.48-0.01n^{\text{3}}+0.31n^{\text{2}}-3.17n+0.12t-0.0058nt \end{array}$$ (4)

Fig 8 shows that the fitted reaction model for calcium hydroxide and Yellow River sediment yielded a high coefficient of determination (*R*^2^ = 0.97), suggesting good fitting performance and better applicability than the corresponding two-dimensional model.

**Fig 8:**
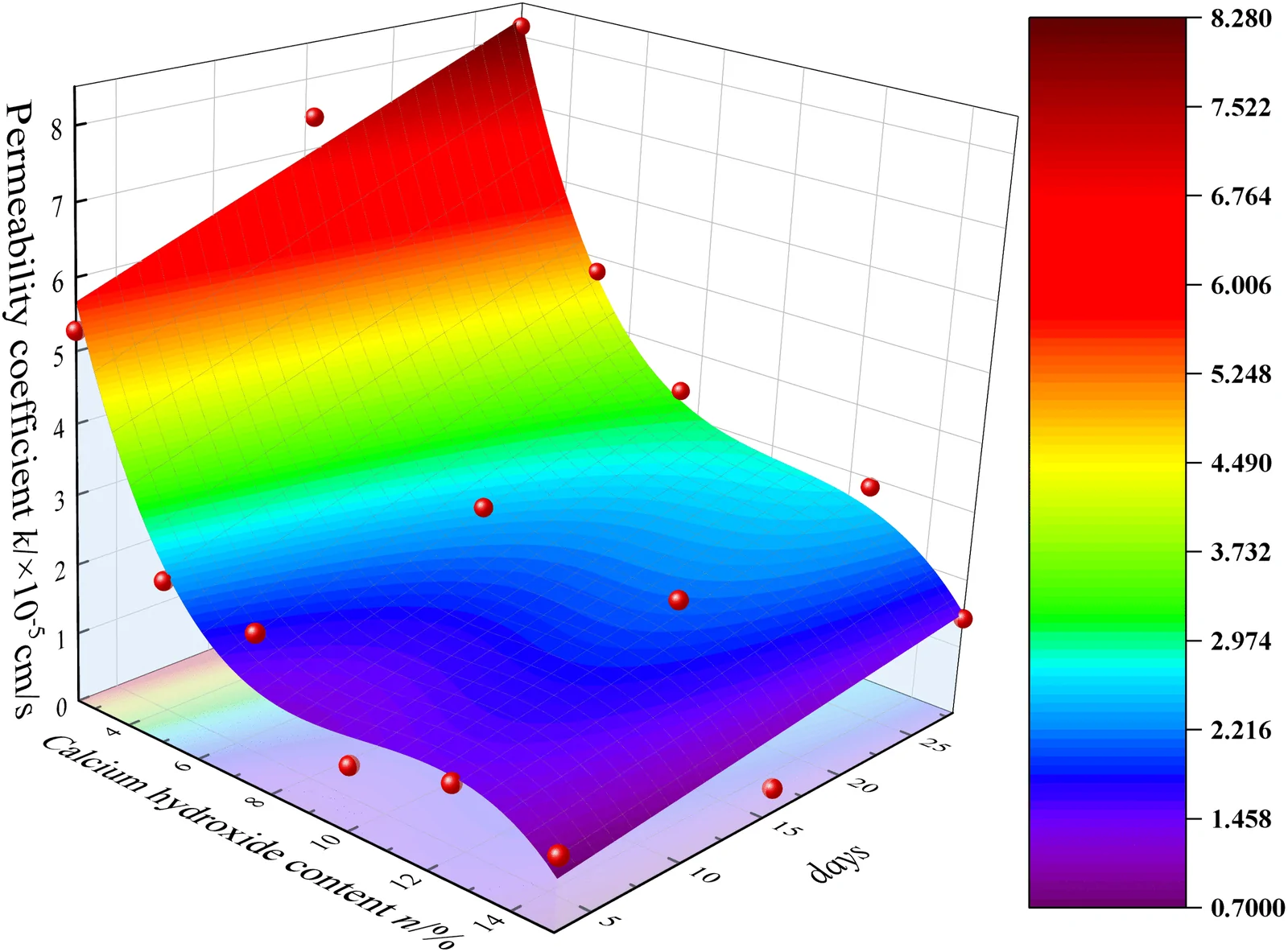
Calcium hydroxide dosing and age multivariate function model.

### 3.2 Analysis of LF-NMR results

#### 3.2.1 Porosity.

Fig 9 shows the porosity variation of Yellow River sediment-based alkali-activated material specimens at different curing ages under different Ca(OH)_2_ dosages. Overall, the specimens exhibited some differences in porosity evolution with curing age, but the overall fluctuation range was relatively limited.

**Fig 9:**
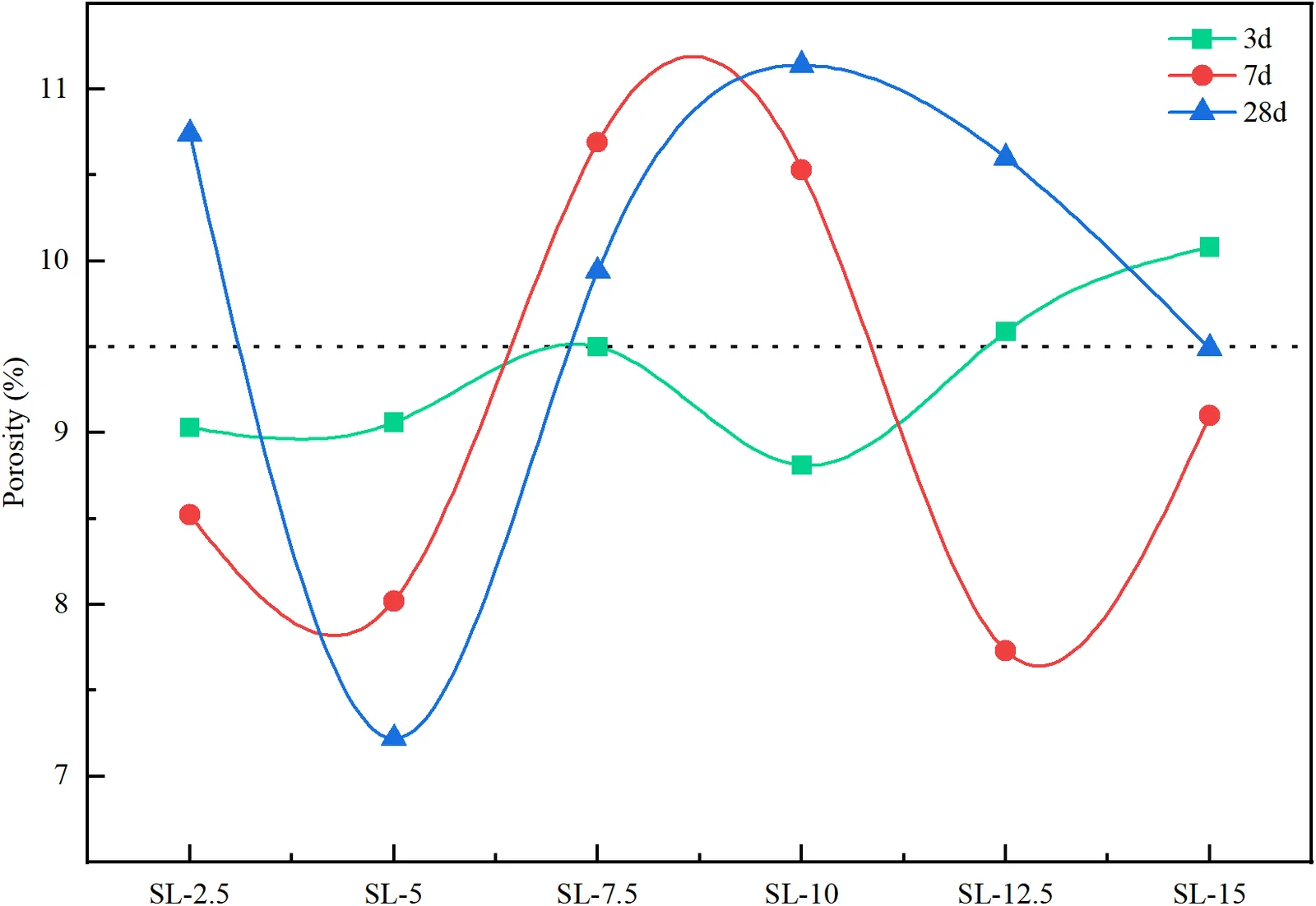
Pore volume of samples under different curing periods.

At a curing age of 3 days, the porosity values of all specimen groups were relatively close to each other and remained within a comparatively stable range. As the curing age increased to 7 and 28 days, the porosity differences among specimens with different Ca(OH)_2_ dosages gradually became apparent, although the porosity values of all specimens still remained mainly around 9.5%. Within the investigated dosage range, the variation of porosity with Ca(OH)_2_ dosage exhibited a non-monotonic pattern at different curing ages, and no significant linear relationship was observed among the specimen groups.

#### 3.2.2 Pore size distribution.

Based on the $T_{\text{2}}$ spectral curves obtained from the nuclear magnetic resonance tests, inverse analysis was performed to characterize the pore size distribution of Yellow River sediment-based alkali-activated material specimens. According to pore size, the pores were classified into micropores (<0.1 μm), small pores (0.1–1 μm), mesopores (1–10 μm), and macropores (10–100 μm) [36].

Fig 10 presents the $T_{\text{2}}$ spectral distributions and corresponding pore size distributions of specimens cured for 3 days under different Ca(OH)_2_ dosages. As shown in Fig 10(a), the spectral curve shape changed significantly with increasing Ca(OH)_2_ dosage, and some specimens gradually shifted from a single-peak-dominated distribution to a multimodal distribution.

**Fig 10:**
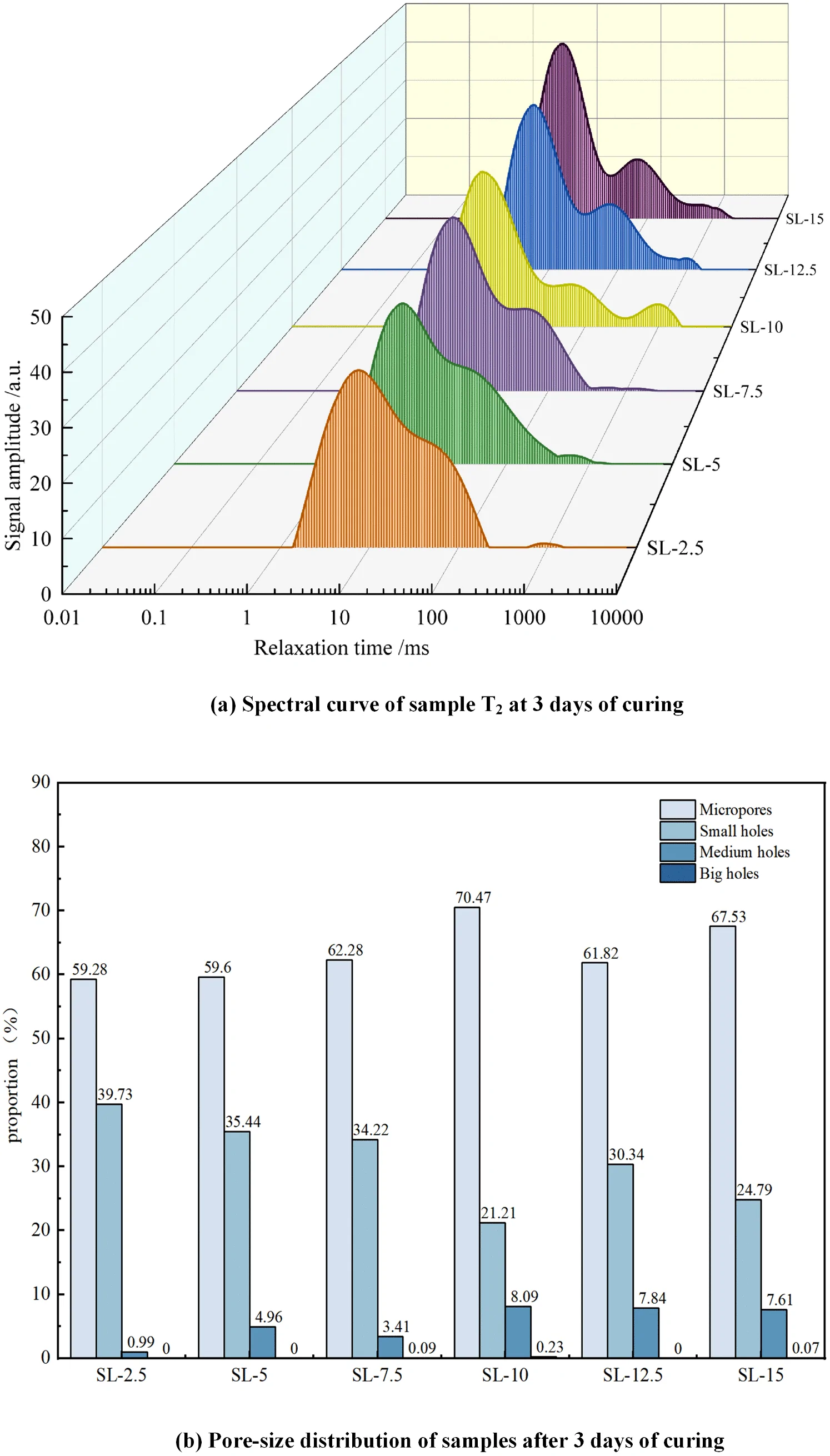
(a) Spectral curve of sample T_2_ at 3 days of curing. (b) Pore-size distribution of samples after 3 days of curing.

Fig 10(b) illustrates the changes in the proportions of pores of different sizes in specimens with different Ca(OH)_2_ dosages. In specimen SL-2.5, the pore structure was mainly composed of micropores, whereas the fractions of small pores and mesopores were low and the fraction of macropores was also limited. With increasing Ca(OH)_2_ dosage to 5.0%, the micropore fraction remained nearly unchanged, the small-pore fraction decreased, and the mesopore fraction increased accordingly. When the dosage was further increased to 15.0%, the micropore fraction increased slightly, the small-pore fraction decreased further, and the mesopore fraction continued to increase, while the macropore fraction changed only slightly overall.

These results indicate that, within the investigated dosage range, the pore size distribution characteristics of the specimens varied significantly with Ca(OH)_2_ dosage, and the proportions of pores at different scales showed inconsistent variation trends with dosage.

The $T_{\text{2}}$ spectral distributions and corresponding pore size distribution results of specimens cured for 7 and 28 days under different Ca(OH)_2_ dosages are shown in Figs 11 and 12, respectively. As shown in Figs 11(a) and 12(a), the overall variation trend of the spectral curves with Ca(OH)_2_ dosage at curing ages of 7 and 28 days was generally consistent with that at 3 days, indicating that the spectral shape changed with dosage.

**Fig 11:**
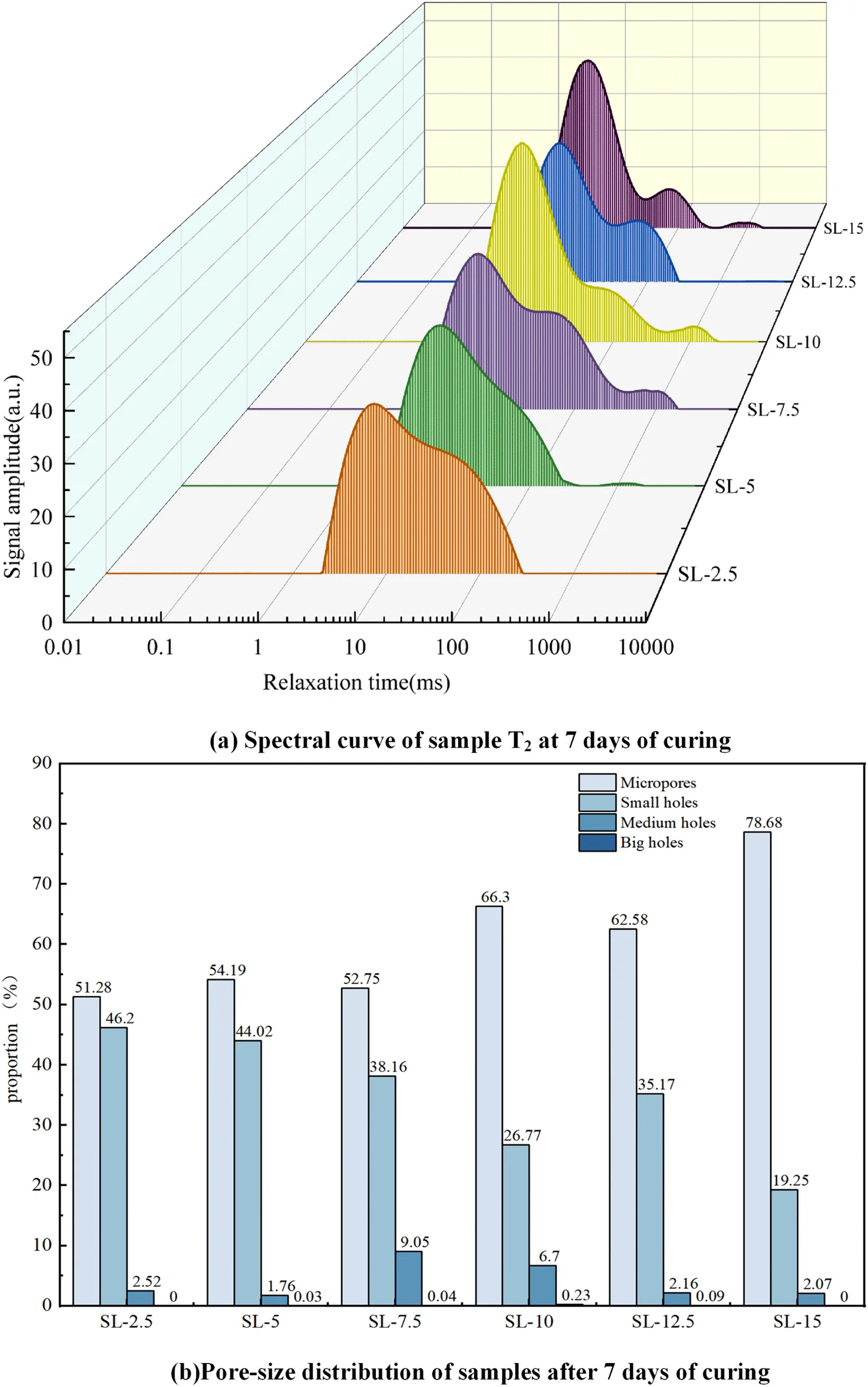
(a) Spectral curve of sample T_2_ at 7 days of curing. (b) Pore-size distribution of samples after 7 days of curing.

**Fig 12:**
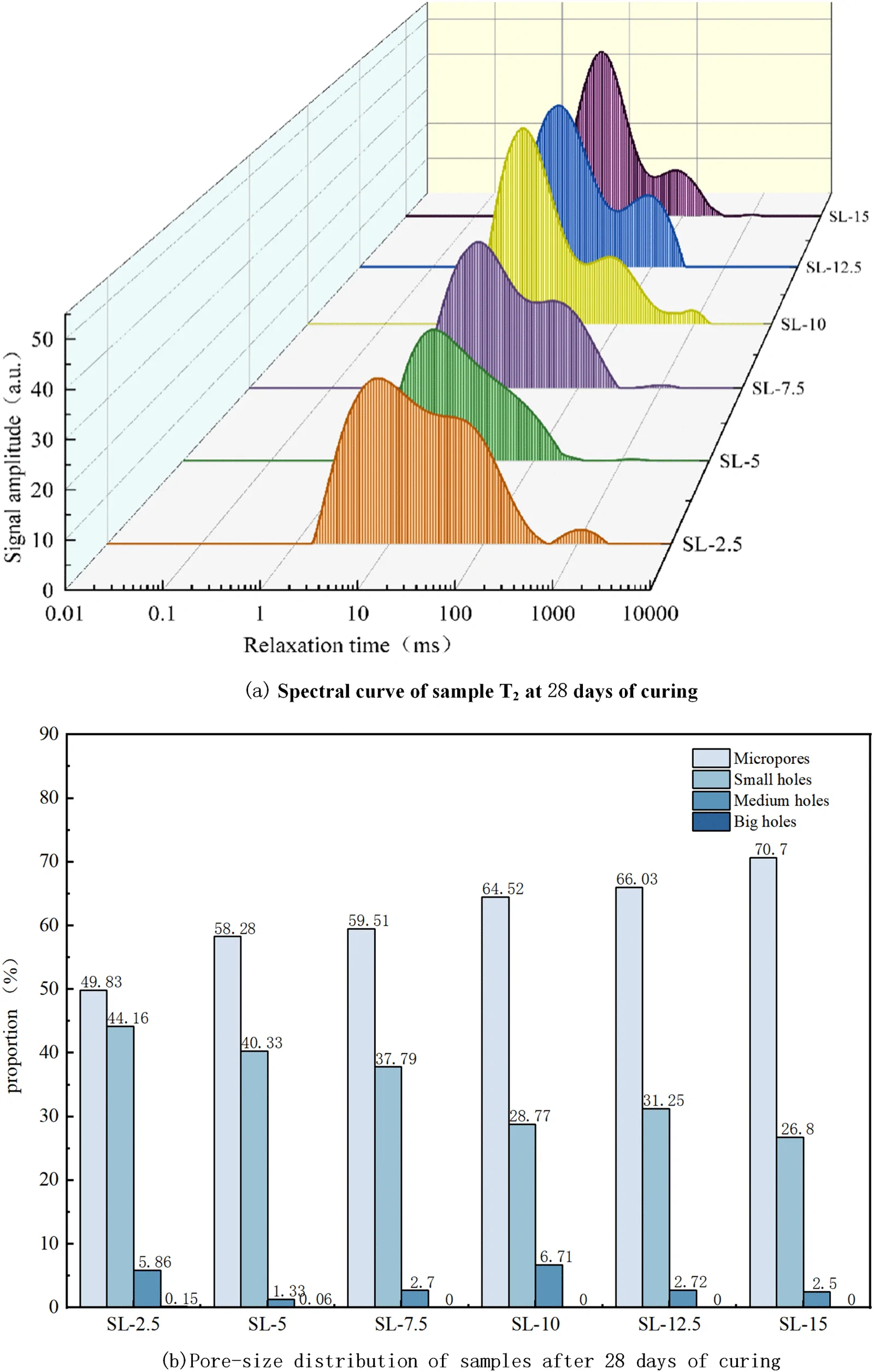
(a) Spectral curve of sample T_2_ at 28 days of curing. (b) Pore-size distribution of samples after 28 days of curing.

Fig 11(b) shows that, at a curing age of 7 days, the pore size distributions of specimens with different Ca(OH)_2_ dosages were markedly different. Compared with the 3-day results, the overall fraction of micropores decreased, while the fractions of small pores and mesopores increased correspondingly, and the fraction of macropores changed only slightly. Increasing Ca(OH)_2_ dosage promoted the formation of micropores at the expense of small pores and mesopores, whereas macropores accounted for only a minor fraction throughout.

At 28 days (Fig 12), the pore size distribution characteristics of specimens with different Ca(OH)_2_ dosages underwent further changes. In the low-dosage specimens, the fraction of micropores was relatively low, while the fractions of small pores, mesopores, and macropores were somewhat higher. As the Ca(OH)_2_ dosage increased from 2.5% to 15.0%, the micropore fraction increased significantly, whereas the fractions of small pores, mesopores, and macropores decreased overall.

Taken together, the results at 3, 7, and 28 days indicate that although the specific proportions of pores at different scales differed among curing ages, the effect of Ca(OH)_2_ dosage on pore size distribution showed good consistency under different curing conditions.

### 3.3 Relationship between microstructure and macroscopic performance

Existing studies have shown that fractal theory can be used to characterize pore structures in geomaterials [37]. According to geometric fractal theory, the cumulative volume fraction ($S_{v}$) of pores with diameters smaller than *r* can be expressed as follows:


$$\begin{array}{c} S_{v}=\frac{r^{3-D}-r_{min}^{3-D}}{r_{max}^{3-D}-r_{min}^{3-D}} \end{array}$$ (5)

where $r_{max}$ and $r_{min}$ are the maximum and minimum pore diameters, respectively, and D is the fractal dimension.

Due to $r_{min}$ is much smaller than $r_{max}$, the above equation can be simplified as follows:


$$\begin{array}{c} S_{v}=\frac{r^{3-D}}{r_{max}^{3-D}} \end{array}$$ (6)

Substituting Eq. (6) into Eq. (5) and taking the logarithm of both sides yields:


$$\begin{array}{c} lgS_{v}=\left(3-D\right)lg\left(T_{\text{2}}\right)-\left(3-D\right)lg\left(T_{2max}\right) \end{array}$$ (7)

where $T_{\text{2}}$ is the transverse relaxation time, and T_2max_ is the maximum transverse relaxation time.

After further simplification, the fractal dimension can be expressed as follows:


$$\begin{array}{c} D=3-K \end{array}$$ (8)

where K denotes the slope of the linear fit between lg(*Sv*) and lg(*T*_2_).

The pore sizes within Yellow River sediment-based alkali-activated materials span a wide range and exhibit a complex distribution, resulting in unsatisfactory fitting performance and making it difficult to characterize the pore structure uniformly using a single fractal dimension. In the pore system, small pores play a dominant role in permeability; therefore, the fractal characteristics of small pores in Yellow River sediment-based alkali-activated materials were investigated in detail.

The fractal dimensions of small pores in specimens at different curing ages are shown in Fig 13. As indicated in Table 6, all correlation coefficients (*R*^2^) were greater than 0.95, indicating strong correlations. At a curing age of 28 days, the variation trend of fractal dimension in Yellow River sediment-based alkali-activated materials was consistent with those at 3 and 7 days, with an inflection point appearing in group SL-12.5, where the fractal dimension tended to become stable. These results suggest that the incorporation of calcium hydroxide accelerated the hydration reaction, leading to increased roughness and tortuosity of the pore surface. In group SL-15, hydration remained relatively active during the curing period from 3 to 28 days, contributing to further optimization of the pore structure. By contrast, the hydration reaction in group SL-12.5 may have been somewhat inhibited, resulting in weaker pore structure evolution.

**Table 6:** Dimension of small pores in samples at different ages.

Age (d)	Constituencies	Fitting results	*D*	*R* ^2^
3	SL-2.5	y=0.22x−0.47	2.78	0.965
	SL-5.0	y=0.20x−0.45	2.80	0.969
	SL-7.5	y=0.19x−0.43	2.81	0.985
	SL-10.0	y=0.11x−0.28	2.89	0.993
	SL-12.5	y=0.17x−0.41	2.83	0.996
	SL-15.0	y=0.13x−0.34	2.87	0.998
7	SL-2.5	y=0.27x−0.60	2.73	0.974
	SL-5.0	y=0.25x−0.53	2.75	0.954
	SL-7.5	y=0.23x−0.55	2.77	0.984
	SL-10.0	y=0.13x−0.33	2.87	0.973
	SL-12.5	y=0.19x−0.43	2.81	0.993
	SL-15.0	y=0.10x−0.22	2.90	0.996
28	SL-2.5	y=0.27x−0.61	2.73	0.979
	SL-5.0	y=0.22x−0.47	2.78	0.952
	SL-7.5	y=0.20x−0.47	2.80	0.985
	SL-10.0	y=0.16x−0.37	2.84	0.992
	SL-12.5	y=0.17x−0.38	2.83	0.999
	SL-15.0	y=0.13x−0.30	2.87	0.990

x = lg(*T*_2_), and y = lg(*Sv*).

**Fig 13:**
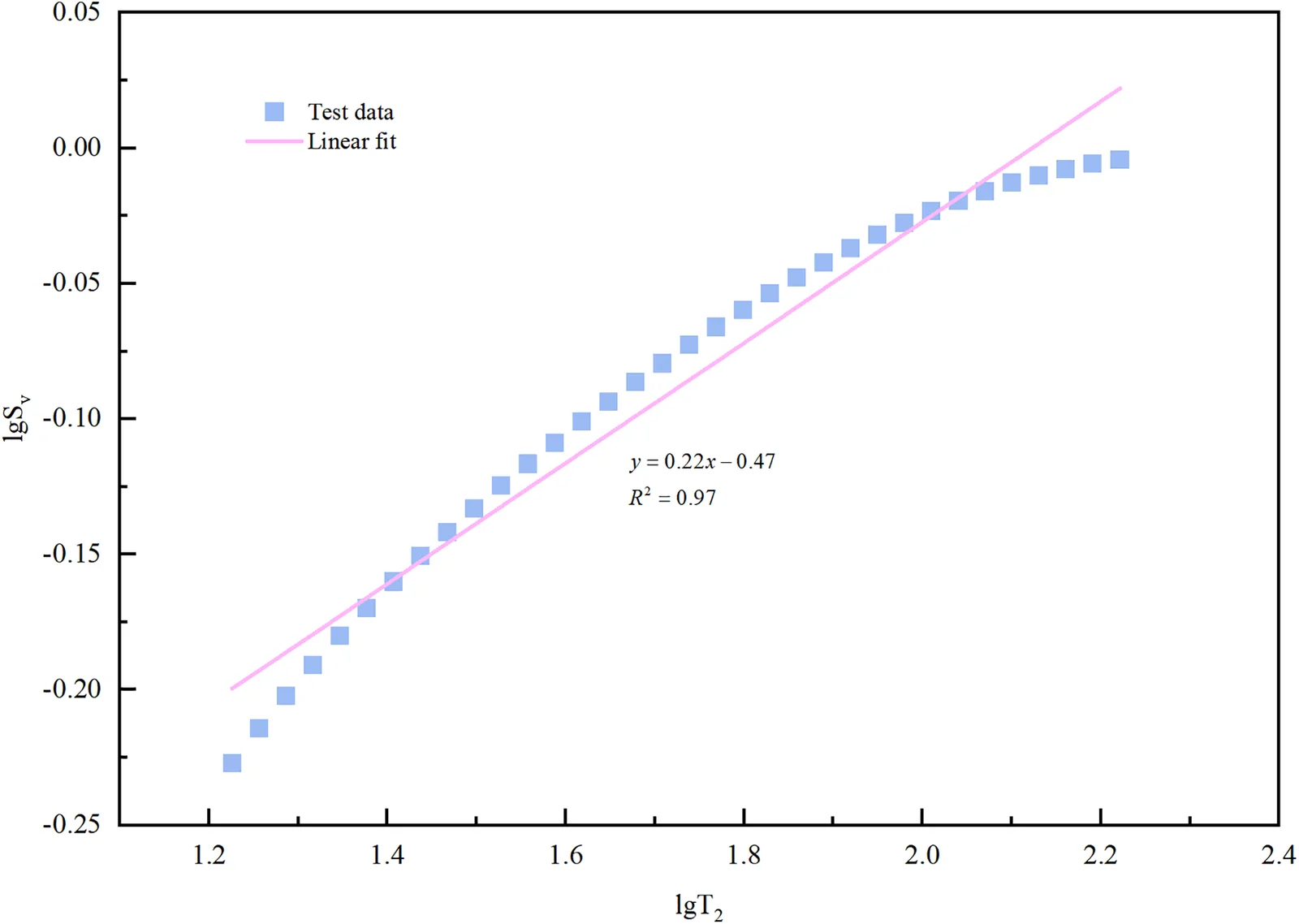
Fractal fitting of the small-pore size distributions.

Group SL-2.5 was not included in this cross-age fitting. In this group the activator dosage was the lowest and the degree of reaction was the most limited, so that the resulting matrix was the least consolidated, its pore network was dominated by loosely bound particles and coarse voids, and its permeability coefficient was considerably higher than that of the other groups. Its structure therefore does not belong to the same structure–property regime as the remaining specimens, and including it would distort the fractal dimension–permeability relationship. A cross-age linear fitting analysis between fractal dimension and permeability coefficient was then conducted for the remaining specimen groups, and the results are shown in Fig 14. The fitted correlation coefficients (*R*^2^) at curing ages of 3, 7, and 28 days were 0.92, 0.96, and 0.92, respectively, indicating a significant statistical correlation between fractal dimension and permeability coefficient. All fitted curves exhibited a monotonic decreasing trend, namely, the permeability coefficient decreased as fractal dimension increased, confirming a negative correlation between pore fractal dimension and permeability. These results suggest that the greater the tortuosity and complexity of the internal pore structure, the better the impermeability of the specimen.

**Fig 14:**
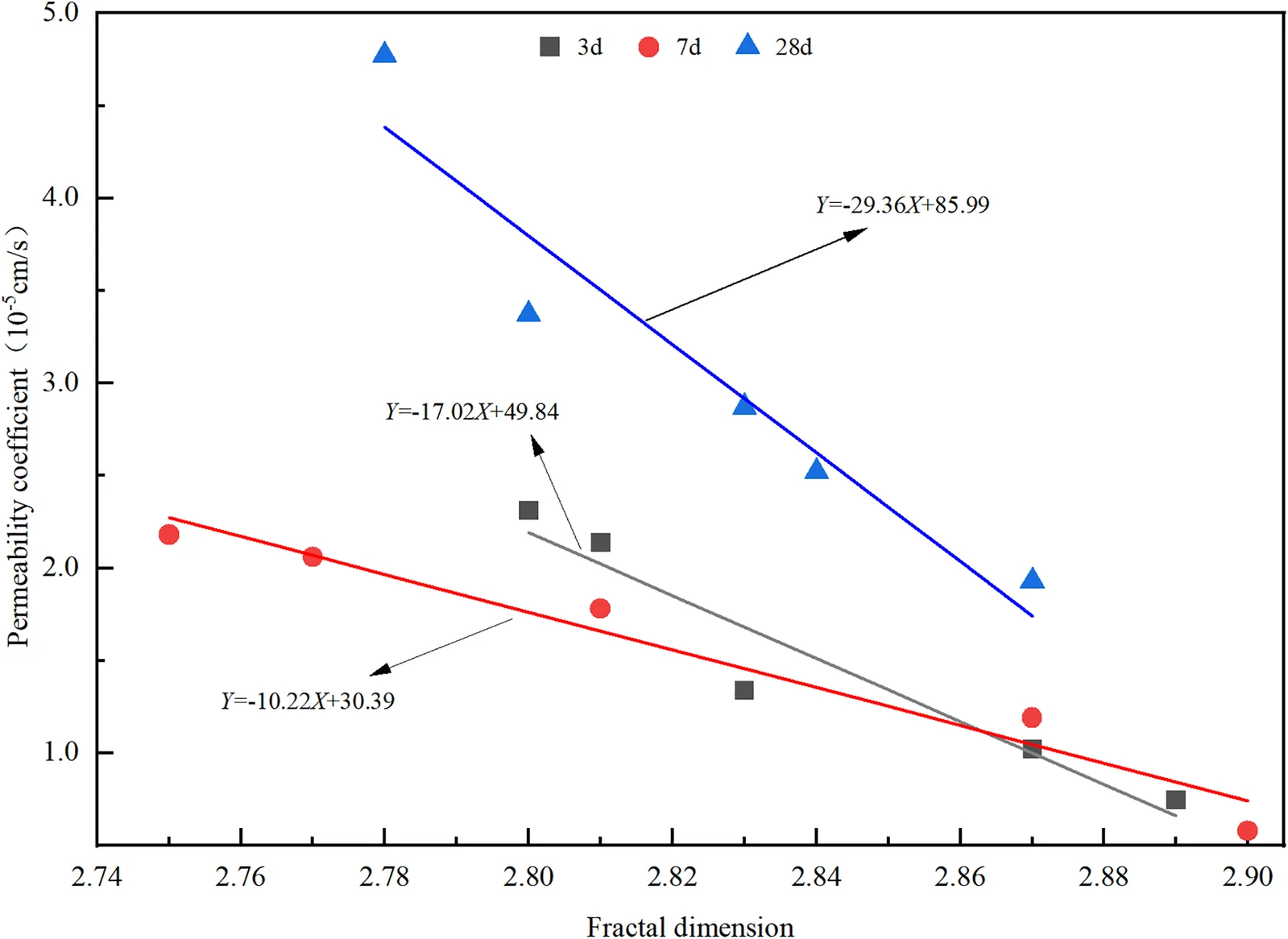
The relationship between permeability coefficient and fractal dimension.

It should be emphasized that the fractal dimension of small pores reflects the overall complexity of the pore structure within a specific scale range, rather than the variation of a single pore size. Therefore, its relationship with permeability should be understood as a statistically meaningful structure–property correlation, rather than a direct causal mapping.

### 3.4 Analysis of engineering case simulation results

In this study, the seepage field is characterized by the distribution of total hydraulic head, and the internal seepage characteristics of the embankment are illustrated by the contour patterns. The numerical results indicate that, compared with the unreinforced condition, the internal seepage field distribution of the embankment changed markedly after reinforcement with Yellow River sediment-based alkali-activated materials. Representative seepage simulation results are presented in Fig 15. To explain the reduction in the overflow (exit) point elevation, the seepage field must be considered as a whole. The reinforcement layer is placed 30 cm beneath the upstream slope surface and has a hydraulic conductivity of 2.52 × 10^−5^ – 2.87 × 10^−5^ cm/s, which is lower than that of the silt-dominated embankment body. This low-permeability layer intercepts part of the seepage entering from the upstream side and forces the remaining flow to pass beneath it, so that the phreatic surface in the downstream part of the embankment is depressed. As a result, the phreatic line intersects the downstream slope at a lower elevation, and the exit point moves from 33.45 m in the unreinforced case to 33.30 m and 33.27 m for the 10.0% and 12.5% cases, respectively, that is, by 0.15 m and 0.18 m, or approximately 2.1% and 2.6% of the embankment height (7.02 m). The unit-width seepage discharge increased only slightly, from 6.14 × 10^−7^ to 6.89 × 10^−7^ and 6.95 × 10^−7^ m³/(s·m), that is, by 12.2% and 13.2%, respectively. Since the discharge is controlled both by the total head difference and by the overall conductance of the flow path, this small increase reflects the redistribution of seepage paths caused by the low-permeability layer rather than a deterioration in the impermeability of the material; the simultaneous lowering of the exit point indicates a downward shift of the downstream phreatic surface under the reinforced conditions, without implying a direct assessment of embankment stability. It should nevertheless be noted that the reduction in the exit point elevation is modest at the scale of the embankment, and that the main value of the numerical analysis lies in demonstrating the consistency between the laboratory-derived permeability parameters and the engineering-scale seepage response rather than in claiming a large improvement in the overall seepage-control capacity.

**Fig 15:**
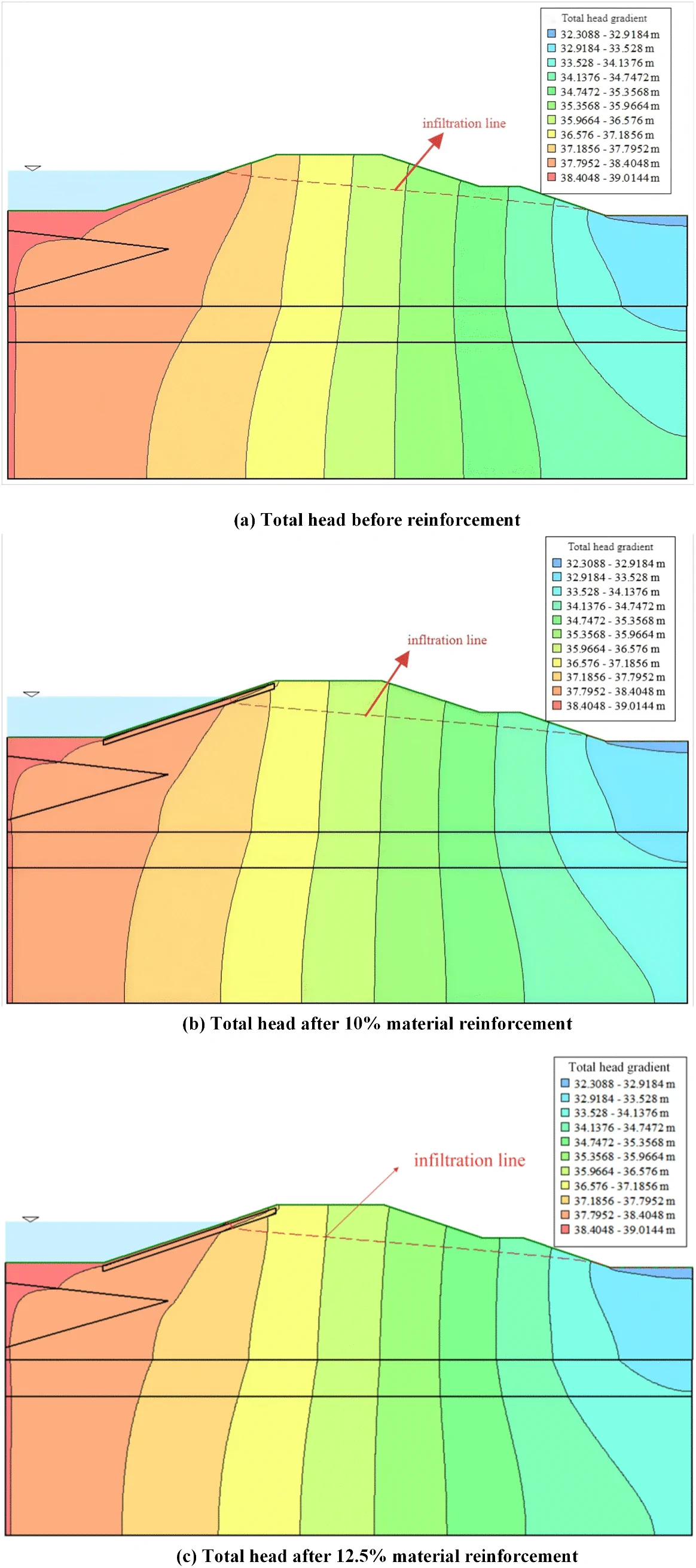
(a) Total head before reinforcement. (b) Total head after 10% material reinforcement. (c) Total head after 12.5% material reinforcement.

Further comparative analysis of the engineering responses under different material dosages revealed that the reinforcement effect varied with dosage. Based on a statistical comparison of engineering indicators, including the elevation of the seepage exit point and the unit-width seepage discharge (Table 7), it was found that when the relatively optimal dosage range identified in the laboratory tests was adopted, both the seepage field distribution and the location of the seepage exit point were improved, resulting in more controllable overall seepage behavior.

**Table 7:** Seepage calculation results.

Operating conditions	Before reinforcement		After reinforcement with a 10% admixture		After reinforcement with a 12.5% admixture	
	Overflow point elevation/m	Single-pass flow rate m³/(s·m)	Overflow point elevation/m	Single-pass flow rate m³/(s·m)	Overflow point elevation/m	Single-pass flow rate m³/(s·m)
Design water level	33.45	6.14 × 10^−7^	33.3	6.89 × 10^−7^	33.27	6.95 × 10^−7^

For completeness, the properties of all six mix designs obtained in the experimental programme are summarized in Table 8. The four dosages that were not used in the numerical simulation are included so that the effect of Ca(OH)_2_ on the performance of the material can be assessed over the whole investigated range.

**Table 8:** Properties of the six mix designs obtained in the experimental programme.

Mix ID	Ca(OH)_2_	k (3 d)	k (7 d)	k (28 d)	D (3 d)	D (7 d)	D (28 d)
SL-2.5	2.5	5.28 ± 1.46	6.78 ± 2.29	8.15 ± 0.12	2.78	2.73	2.73
SL-5.0	5.0	2.31 ± 0.41	2.18 ± 0.84	4.78 ± 0.21	2.80	2.75	2.78
SL-7.5	7.5	2.14 ± 0.18	2.06 ± 0.10	3.37	2.81	2.77	2.80
SL-10.0	10.0	0.92	1.19 ± 0.46	2.52 ± 0.15	2.89	2.87	2.84
SL-12.5	12.5	1.32	1.77 ± 0.12	2.87 ± 0.83	2.83	2.81	2.83
SL-15.0	15.0	1.02 ± 0.20	0.56 ± 0.13	1.43	2.87	2.90	2.87

k is given in ×10^−5^ cm/s. D is the fractal dimension of the small pores (0.1–1 μm).

These results indicate that the material mix proportions determined in the experimental stage through permeability testing and pore structure analysis also showed reasonable applicability at the engineering scale, thereby supporting the relevance of the experimental findings to practical engineering conditions. The variation in unit-width seepage discharge under different conditions was relatively small, and this variation mainly reflected the redistribution of seepage paths under reinforced conditions, rather than deterioration in the impermeability of the material.

Overall, the engineering simulation results suggest that the permeability parameters derived from the experimental stage can capture certain differences in seepage response at the engineering scale, indicating that the permeability variation pattern obtained at the laboratory scale retains a degree of trend consistency in the engineering-scale model. It should also be noted that the unit-width seepage discharge varied only slightly under different conditions and did not exhibit a pronounced reduction. Therefore, the main value of the engineering validation in this study is to assess the rationality of the material parameter response in the engineering model, rather than to directly prove a significant enhancement in overall seepage-control capacity. The long-term engineering applicability of the material still requires further study considering construction disturbance, service environment, and durability.

### 3.5 Limitations and future work

Several constraints of this study should be acknowledged. The sediment was collected from a single reach of the Yellow River at Huayuankou, so its gradation, mineralogy and reactivity are representative of that location only; sediment from other reaches may differ, and the optimum dosage identified here should not be transferred to other sources without local verification. Only the Ca(OH)_2_ dosage was varied. The NaOH dosage, water content, moulding density and curing conditions were held constant, so the interactions between the calcium source and the other mix-design parameters remain untested. The curing ages were limited to 3, 7 and 28 days, which is short relative to the service life of an embankment; whether the permeability continues to change beyond 28 days, and how the material behaves under wetting–drying cycles, frost and construction disturbance, has not been established.

The pore structure was characterized by LF-NMR alone. LF-NMR detects the hydrogen-bearing fluid confined in the pore space and therefore reflects the size distribution of water-filled pores, but it does not resolve pore connectivity or the geometry of individual pores. Complementary techniques such as mercury intrusion porosimetry, gas adsorption and scanning electron microscopy, together with X-ray diffraction for phase identification, would be needed to confirm the reaction path proposed in Section 3.1.2. The explanation given there is a reasoned interpretation of the macroscopic and NMR results supported by the literature, not a directly demonstrated mechanism.

The numerical analysis has its own limits. Permeability coefficients measured on small laboratory specimens were applied directly to a two-dimensional embankment model, without accounting for scale effects, construction-induced heterogeneity or the spatial variability of the foundation soils, whose distribution was simplified into six zones. No formal uncertainty analysis was carried out, and no independent data set was available to validate the dosage–age–permeability relationship, which should therefore be regarded as an empirical description of the range investigated here rather than as a predictive model. The simulation reproduces the direction of the seepage response rather than the absolute discharge, and field monitoring at full scale would be required before the material is used in practice.

## 4. Conclusion

Yellow River sediment activated with a NaOH–Ca(OH)_2_ composite was tested at six Ca(OH)_2_ dosages and three curing ages, and the measured permeability was related to the pore structure resolved by low-field NMR before being carried over to an embankment-scale seepage model. Permeability proved far more sensitive to dosage than to curing time. Raising Ca(OH)_2_ from 2.5% to 10.0% reduced the permeability coefficient by 82.58%, 82.45% and 69.07% at 3, 7 and 28 days, whereas raising it further to 12.5% gave back part of that gain, with increases of 43.48%, 48.70% and 13.80%; at 15.0% the coefficient fell again. The permeability response was therefore non-monotonic, with a temporary rebound at 12.5% and a further decrease at 15.0%; the small-pore fractal dimension turns over at 12.5% as well, being lower there than at 10.0% and 15.0% at every age.

Curing age showed no comparable regularity. SL-5.0, SL-7.5 and SL-15.0 first became less permeable and then more permeable, while SL-2.5, SL-10.0 and SL-12.5 became steadily more permeable up to 28 days. Prolonging curing is therefore not by itself a dependable way of lowering permeability in this system, and the age effect has to be read together with the activator dosage.

The NMR results point to the pore network rather than to the amount of pore space. Total porosity stayed close to 9.5% for every mix at every age, yet the permeability coefficient spanned almost an order of magnitude, so a change in total void volume cannot explain the differences observed. What changed was how that volume is distributed: the fractal dimension of the small pores (0.1–1 μm) correlated negatively with the permeability coefficient at all three ages (R² = 0.92, 0.96 and 0.92), and a multivariate fit of permeability against dosage and age reached R² = 0.97. The least permeable specimens were those with the most tortuous small-pore network, whatever their total porosity.

At the embankment scale the laboratory parameters reproduced the direction of the change. A 70 cm reinforcement layer placed 30 cm below the upstream slope lowered the seepage exit point from 33.45 m to 33.30 m at 10.0% and 33.27 m at 12.5%, reductions of 0.15 m and 0.18 m, while the unit-width discharge moved from 6.14 × 10 ⁻ ⁷ to 6.89 × 10 ⁻ ⁷ and 6.95 × 10 ⁻ ⁷ m³/(s·m). The discharge rose slightly instead of falling, which shows that the layer diverts part of the flow rather than sealing it; the gain at this scale is the lower phreatic surface, not a large cut in total seepage. The dosage–age–permeability relationship is an empirical description of the range investigated here rather than a predictive model, and the numerical analysis reproduces trends rather than absolute discharges.
